# Characterization of a yeast interfering RNA larvicide with a target site conserved in the *synaptotagmin* gene of multiple disease vector mosquitoes

**DOI:** 10.1371/journal.pntd.0007422

**Published:** 2019-05-20

**Authors:** Keshava Mysore, Ping Li, Chien-Wei Wang, Limb K. Hapairai, Nicholas D. Scheel, Jacob S. Realey, Longhua Sun, Joseph B. Roethele, David W. Severson, Na Wei, Molly Duman-Scheel

**Affiliations:** 1 Indiana University School of Medicine, Department of Medical and Molecular Genetics, South Bend, IN, United States of America; 2 The University of Notre Dame Eck Institute for Global Health, Notre Dame, IN, United States of America; 3 The University of Notre Dame Department of Civil and Environmental Engineering and Earth Sciences, Notre Dame, IN, United States of America; 4 The University of Notre Dame Department of Biological Sciences, Notre Dame, IN, United States of America; 5 The University of the West Indies, Department of Life Sciences, St. Augustine, Trinidad, Trinidad and Tobago; Centers for Disease Control and Prevention, UNITED STATES

## Abstract

New mosquito control strategies are vitally needed to address established and emerging arthropod-borne infectious diseases. Here we describe the characterization of a yeast interfering RNA larvicide that was developed through the genetic engineering of *Saccharomyces cerevisiae* (baker’s yeast) to express a short hairpin RNA targeting the *Aedes aegypti synaptotagmin (Aae syt)* gene. The larvicide effectively silences the *Aae syt* gene, causes defects at the larval neural synapse, and induces high rates of *A*. *aegypti* larval mortality in laboratory, simulated-field, and semi-field trials. Conservation of the interfering RNA target site in multiple mosquito species, but not in humans or other non-target species, suggested that it may function as a broad-range mosquito larvicide. In support of this, consumption of the yeast interfering RNA larvicide was also found to induce high rates of larval mortality in *Aedes albopictus*, *Anopheles gambiae*, and *Culex quinquefasciatus* mosquito larvae. The results of these studies suggest that this biorational yeast interfering RNA larvicide may represent a new intervention that can be used to combat multiple mosquito vectors of human diseases.

## Author summary

It is critical that we develop new strategies for the environmentally safe control of disease vector mosquitoes. In this study, baker’s yeast was genetically engineered to produce interfering RNA molecules corresponding to the mosquito *synaptotagmin (syt)* gene, but which do not match any genes in humans or other non-target organisms. Larval consumption of the yeast, which was prepared in a ready-to-use dried inactivated tablet formulation, turned off the *syt* gene and disrupted function of the mosquito nervous system. The yeast larvicide induced high levels of larval mortality in a variety of different human disease vector mosquito species. Inactivated yeast interfering RNA tablets performed well in contained semi-field experiments, suggesting that this technology, if further developed and optimized in the field, could one day be used for the biorational control of mosquitoes and the prevention of multiple mosquito-borne illnesses.

## Introduction

Larviciding, the application of microbial or chemical agents to kill mosquito larvae in aquatic habitats, is a key component of integrated mosquito control and disease prevention strategies. *Aedes* mosquitoes, the primary vectors of dengue, Zika, yellow fever, and chikungunya viruses, lay eggs in water-filled containers located within or close to human dwellings in urban settlements and are therefore susceptible to larvicides [1]. Likewise, larviciding is a priority for control of *Culex pipiens* complex mosquitoes [2], the principle vectors of lymphatic filariasis [3] and West Nile virus [2]. The 1999 introduction and subsequent spread of West Nile virus across the continental United States [2] has sparked high interest in the development of new environmentally-friendly products for controlling *Culex* mosquitoes. Larvicide treatments are often targeted toward catch basins, major sources of *Culex* mosquitoes in urban areas and a primary focus of many mosquito abatement districts in the U.S. [4]. Due to the increased observation of insecticide resistance to existing larvicides and escalating concerns for adverse effects of pesticides on non-target species, new larvicidal agents are vitally needed to address existing as well as emerging mosquito-borne diseases [5]. Tools that can be used to target multiple vector mosquito species are of particular interest.

Although RNA interference (RNAi) has been applied for functional characterization of mosquito genes, this approach, which is attracting attention in the agricultural pest control community [6], is still a largely unexplored approach for control of disease vector mosquitoes. The short length (21–25 bp) of custom small interfering RNAs (siRNAs) and their short hairpin RNA (shRNA) counterparts permits the design of interfering RNA that recognizes target sites conserved in disease vector mosquitoes, but which are not found in non-target organisms. We recently began to pursue screens for siRNA larvicides that target *A*. *aegypti* [7] and *Anopheles gambiae* [8] mosquito larvae. Through characterization of these interfering RNA molecules, we hope to develop an arsenal of interfering RNA larvicides which can be used to combat resistance that might arise from point mutations in any single target sequence [7, 8].

To facilitate the cost-effective production of interfering RNA and delivery of RNA pesticides to mosquitoes in the field, we recently began to engineer *Saccharomyces cerevisiae* (baker’s yeast), a model organism that is genetically tractable and inexpensive to culture, to produce shRNA corresponding to select genes/target sequences identified in the siRNA larvicide screens [7, 8]. The use of *S*. *cerevisiae* for larvicide delivery has numerous advantages. First, yeast is a strong odorant attractant and a source of nutrition for laboratory-bred mosquito larvae [9]. Thus, the shRNA delivery system simultaneously serves directly as larval bait. Our laboratory studies have also demonstrated that yeast interfering RNA tablets attract gravid female mosquitoes to lay eggs in treated containers [7], offering the advantage of a lure-and-kill system. Additionally, interfering RNA is generated through yeast culturing, significantly reducing RNA production costs, and yeast production can be readily scaled for commercialization purposes. Furthermore, yeasts have been cultivated worldwide for thousands of years, and this technology can be adapted to resource-limited countries with constrained infrastructures. Dried yeast can be packaged and shipped in both active (live) and inactive (dead) forms, which can facilitate regional production and distribution. *S*. *cerevisiae*, a natural product that is often used in food and alcoholic beverage preparation and sold as a dietary supplement, is non-toxic. Finally, yeast interfering RNA larvicides can be heat-inactivated, which is desirable from an environmental standpoint, and prepared into a ready-to-use tablet formulation that can be integrated into existing *Aedes* mosquito control programs [7, 10, 11].

Here we describe characterization of a yeast interfering RNA larvicide with a target site in the *Aae syt* gene. Syt is an evolutionarily conserved calcium binding protein which functions as a calcium sensor that regulates neurotransmitter release at neural synapses (reviewed in [12]). The interfering RNA target site in the *A*. *aegypti syt* gene is conserved in multiple species of mosquitoes, including *Aedes*, *Anopheles* and *Culex* mosquito species, but not humans or other non-target species, suggesting that it may function as a broad-based mosquito larvicide. The results of these studies suggest that this yeast interfering RNA larvicide may represent a new intervention that can be used to combat multiple mosquito vectors of human diseases.

## Methods

### Mosquito rearing

*A*. *aegypti* Liverpool-IB12 (LVP-IB12) strain mosquitoes, *A*. *albopictus* (Gainesville strain from BEI Resources), *A*. *gambiae* G3 strain mosquitoes (from BEI resources), and *C*. *quinquefasciatus* JHB strain (from BEI Resources) mosquitoes were used. Mosquitoes were reared as described previously [13], except that commercially purchased sheep blood (HemoStat Laboratories, Dixon, CA) was delivered to adult females through an artificial membrane feeding system. Insects were reared in an insectary maintained at 26°C, at ~80% humidity, and under a 12 hr light/12 hr dark cycle with 1hr crepuscular periods at the beginning and end of each light cycle.

### Larval soaking experiments

Soaking experiments were performed in conjunction with larval lethal siRNA screens [7, 8]. Custom siRNAs corresponding to the following target sequences were purchased from Integrated DNA Technologies (IDT) for use in the screen:

#427: 5’ AUUAUUAGGUUCAGCAUACAA3’ in *syt (AAEL000704);* Control sequence which is not present in any of the mosquito species: 5’GAAGAGCACUGAUAGAUGUUAGCGU3’ [14]. As discussed previously [7, 8, 15], larval soaking screens were performed in duplicate according to the method of Singh et al. [15], with 20 L1 larvae soaked for four hours in 0.5 μg/μl siRNA. After soaking treatments, larvae were reared and assessed as described in the WHO [16] larvicide testing guidelines. Screen data were assessed using the Fisher’s exact test.

### Generation of yeast interfering RNA larvicide strains and yeast culturing

shRNA-encoding DNA oligonucleotides corresponding to the #427 target sequence were custom synthesized by Invitrogen Life Technologies. As discussed in Hapairai et al. [7], both transient as well as stably transformed yeast strains expressing shRNA #427 were generated using methodology previously used to generate control shRNA expression strains, which were also used in this study. For transient transformations, the #427 shRNA expression cassette was cloned into *pRS426 GPD*, a non-integrating bacteria-yeast shuttle vector with a *URA3* selection marker; this facilitated constitutive expression of #427 shRNA, which was placed under control of a strong constitutively active *GPD* promoter [17]. After confirming the inserts through restriction digesting and sequencing, *S*. *cerevisiae* strain *BY4742* [18], genotype *MATα his3Δ1 leu2Δ0 lys2Δ0 ura3Δ0*, was transformed with the plasmid. Positive transformants were selected on SC minimal media lacking uracil. Please see Mysore et al. [10] for a detailed description of this methodology.

**Fig 1:**
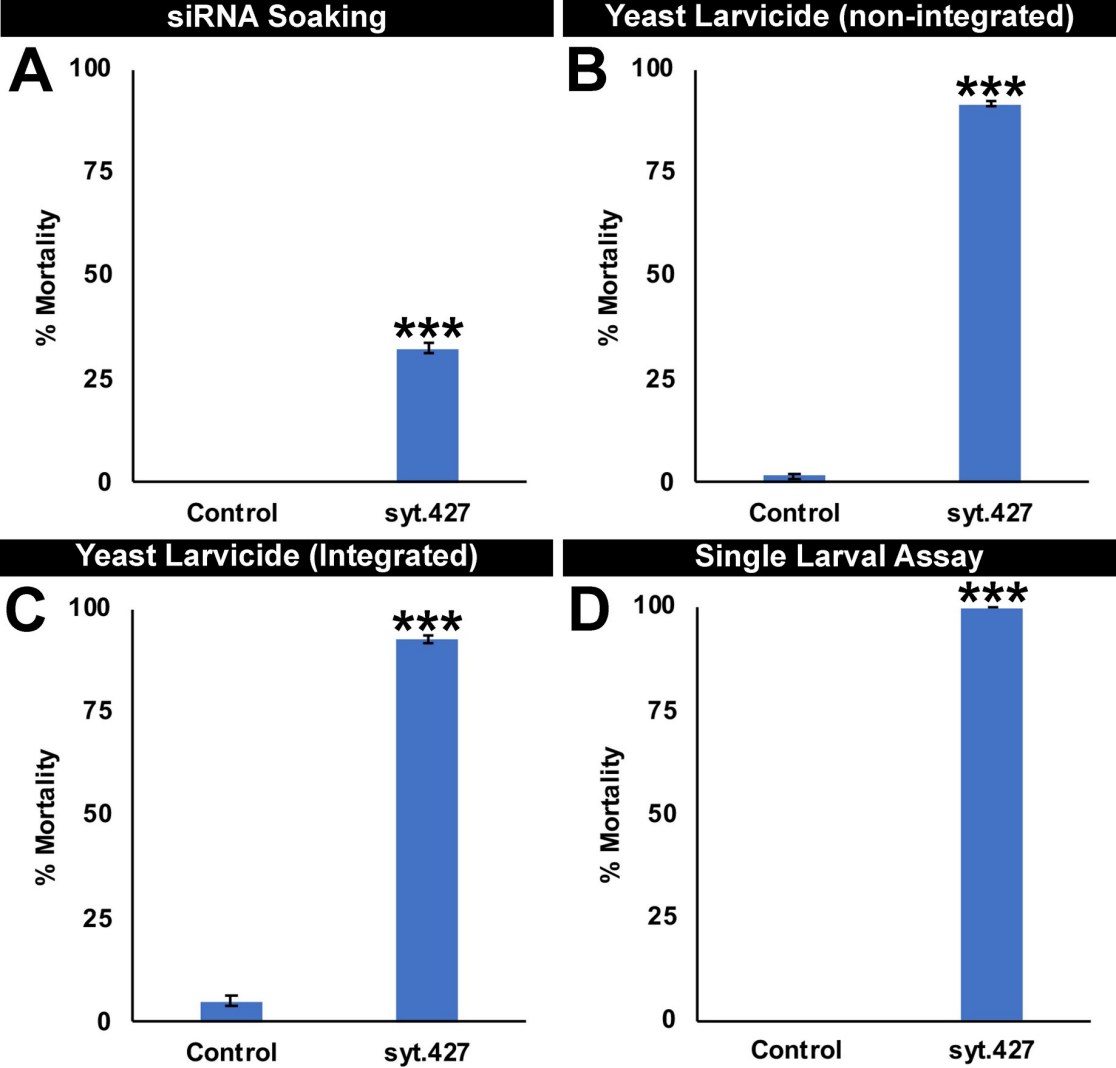
Larval mortality induced by interfering RNA larvicides targeting *Aae syt*. siRNA larvicide #427 was identified in soaking screens for mosquito larval lethal genes. Although control siRNA-treated larvae lived, significant larval mortality was observed in larvae soaked in siRNA #427 (A; experiments were performed in duplicate with 20 larvae/treatment, and data were analyzed with the Fischer’s exact test). shRNA corresponding to the #427 target sequence or to a control sequence corresponding to no *A*. *aegypti* genes was expressed in *S*. *cerevisiae* from a plasmid (B) or from shRNA expression cassettes integrated into the *S*. *cerevisiae* genome (C, D). *A*. *aegypti* larval consumption of inactivated dried yeast interfering RNA tablets prepared from either strain resulted in significant larval mortality (B, C, D; data were analyzed with a t-test). Data shown in B were compiled from multiple replicate experiments (each with 20 larvae/container) conducted on a total of 240 larvae/condition; data shown in C were compiled from multiple replicate experiments (each with 20 larvae/container) conducted on a total of 300 larvae/condition. In panel D, larvae were reared as individuals; results compiled from three biological replicate experiments (each with 20 individual larvae/condition) were analyzed with a t-test. In panels A-D, data are represented as mean percentage mortality, and error bars represent standard errors of the mean (SEM). *** = P < 0.001 in comparison to control-treated larvae. Yeast bearing stably integrated shRNA expression cassettes (C, D) were used in all subsequent studies/figures in this investigation.

For generation of stable transformants (which were subsequently used in all assays with the exception of Fig 1A and 1B), DNA encoding the #427 shRNA was ligated downstream of the strong inducible *Gal1* promoter [19, 20] and upstream of the *cyc1* terminator as described [7]. The resulting *Gal1* promoter-shRNA-*cyc1* terminator expression cassettes were inserted into the multiple cloning sites of yeast integrating plasmid shuttle vectors *pRS404* and *pRS406* [21], which have *TRP1* and *URA3* selection markers, respectively. The resulting plasmids facilitated integration of two copies of the #427 shRNA expression cassette at both the *trp1* and *ura3* loci of the *S*. *cerevisiae CEN*.*PK* strain, which has the following genotype: *MAT*a/α *ura3-52/ura3-52 trp1-289/trp1-289 leu2-3_112/leu2-3_112 his3* Δ*1/his3* Δ*1 MAL2-8C/MAL2-8C SUC2/SUC2* [22]. Growth on synthetic complete media lacking tryptophan or uracil facilitated the selection of stable transformants. PCR and sequencing further verified integration events at both loci. Following strain generation, syt.427 and control interfering RNA strains (referred to as control) were cultured as described [7]. Galactose inductions were performed as discussed previously [7]. Dried inactivated yeast interfering RNA tablets were prepared as described [10]. Further technical information is provided in our detailed protocol [10].

### Larvicide assays

#### Laboratory trials

Laboratory larvicide bioassays were performed in the insectary as described [8] and conformed to the WHO larvicide testing guidelines [16]. Larvae fed with inactivated syt.427 or control interfering RNA larvicide tablets were assessed in parallel. Results were compiled from multiple biological replicate experiments, each with at least three replicate containers per condition. In each replicate container assay, 20 larvae were fed with a single control or syt.427 yeast tablet at the start of the assay, which allowed larvae to feed on the yeast *ad libitum* throughout the experiment. 150 μL of 6% w/v liver powder (MP Biomedicals) in distilled water was provided as a dietary supplement to L4 larvae as discussed previously [10]. A t-test was used for analysis of these laboratory trials. Dose-response curves were generated as described previously [7] through conduction of three biological replicate experiments, each with four replicate containers. For assessment of dose-response curve data, Abbot’s formula was used to account for <2% mortality in control larvae as described [16]. Data from all replicate experiments were pooled for analysis, and LD_50_ values with 95% confidence intervals were calculated using SPSS software and a log dosage-probit mortality regression line.

To further assess syt.427-treated survivors, 200 L1 larvae placed in 1 L of water were fed 10 yeast tablets prepared from *CEN*.*PK* (no shRNA expression construct), control interfering RNA (which expresses the control shRNA expression construct), or syt.427 strains that were prepared as described [10]. Three biological replicate experiments, each with three 1 L containers bearing 200 larvae, were performed. Following treatment, 15 female adult survivors from each of three biological replicate containers were mated to untreated adult males that had been reared under standard lab culturing conditions as described [13]. Each pair of mosquitoes was maintained on 10% sucrose solution. Following delivery of a blood meal to the female, pairs were left to mate for three days, and females laid eggs for two days. For each individual female, the number of eggs laid (fecundity) and the number of larvae hatched from these eggs (fertility) was recorded. Fecundity data combined from three replicate experiments were evaluated with ANOVA. For evaluation of fertility data, the percentage hatch rates from three biological replicate experiments were transformed to arcsin(sqrt(x)) before analysis of the data with ANOVA. To assess longevity, in each of two biological replicate experiments, 200 L1 larvae were treated with 10 CEN.PK, control interfering RNA, or syt.427 yeast tablets prepared as described [7, 10], and 15 female adult survivors were maintained in a cage. Individual deaths were monitored and recorded on a daily basis for 35 days in each of two biological replicate experiments. Longevity data compiled from the two replicate experiments were analyzed using Kaplan-Meier analysis and a log-rank test.

#### Individual rearing/feeding experiments

In addition to assays performed on groups of 20 larvae (see above), individual larvae were subjected to larvicide treatment. For assays on individual larvae, 4 mg yeast pellets were prepared by pelleting 5 mL of stably transformed control or syt.427 yeast cultured as described previously [10]. Pellets were then processed and dried as described [10] except that the pellet drying time was reduced to one day. Each individual larva was then placed in a container filled with 50 mL distilled water and a mini yeast pellet. 10 μL of 6% w/v liver powder in distilled water was also provided on day one, and 20 μL of 6% w/v liver powder was provided on days two and three. 20 individual control- or syt.427-treated individual larvae were assessed in each biological replicate experiment. Data from three biological replicate experiments were combined and assessed through Chi-square analysis. This methodology was employed only for collection of the results shown in Fig 1D.

#### Simulated-field trials

Experiments were performed and analyzed as described above, but with two variations. In one set of experiments, larvicide trials were conducted using *A*. *aegypti* LVP-IB12 larvae that were tested in 100 mL of rainwater (rather than sterile distilled water) collected in South Bend, IN during summer 2018. The water, which was collected from the roof, initially contained dirt and leaves that were removed through filtration through a 100 micron filter. In another set of experiments, the larvae used were hatched from the F2 generation of an *A*. *aegypti* strain generated from eggs collected in ovitraps in Trinidad, Trinidad and Tobago; these larvae were evaluated in distilled water.

#### Semi-field trials

Semi-field trials were conducted using LVP-IB12 strain mosquitoes on an outdoor rooftop laboratory in Notre Dame, IN during July and August 2018. These experiments conformed to the WHO [16] larvicide testing guidelines and were performed in tents that prevented entrance or exit of living creatures from the experimental site. 30 L containers with a depth of 46 cm contained 26 L of water, 20 larvae, and one yeast pellet (prepared from control or syt.427 yeast as described previously [10]) and were covered with mesh to provide a second layer of containment. Three biological replicate experiments with three replicate containers per condition were performed. Temperatures ranged from 13.5°C to 42.0°C during the testing period, with a mean daytime temperature of 27°C and a mean nighttime temperature of 23°C. Humidity levels averaged 68±18% during the trial period.

### Whole mount *in situ* hybridization and immunohistochemistry

A riboprobe corresponding to *Aae syt* was synthesized according to the Patel [23] protocol and used for *in situ* hybridization experiments conducted on L4 brains. Living L4 larvae were fixed for *in situ* hybridization experiments, which were performed in triplicate as described previously [24]. Following mounting and imaging of tissues with a Zeiss Axioimager equipped with a Spot Flex camera, mean gray values (average signal intensity over the selected area) were calculated using FIJI ImageJ software for digoxigenin-labeled transcript signals in control or experimental brains; in these studies, data were combined from three replicate experiments. A paired t-test was used to statistically analyze transcript quantification data.

Immunohistochemical staining experiments were performed in triplicate as previously described [25, 26] using mAb nc82 anti-Bruchpilot [27] (DSHB Hybridoma Product nc82, which was deposited by E. Buchner to the DSHB) and TO-PRO-3 iodide (Molecular Probes, Eugene, OR). For each of the three biological replicate experiments, larvae from four replicate containers per condition were fixed, processed, and evaluated. Following immunohistochemical processing, tissues were mounted and imaged using a Zeiss 710 confocal microscope and Zen software. These images were analyzed through use of FIJI ImageJ and Adobe Photoshop CC 2018 software. For antibody staining intensity analyses, mean gray values were calculated as described [28] for brains combined from the three replicate experiments. Data were statistically analyzed using a paired t-test.

#### Toxicity studies in non-target species

*D. melanogaster*: The survival of *D*. *melanogaster* larvae that fed on syt.427 or control interfering RNA yeast was evaluated. Larvae from the wild type Oregon R stock [29] were used in these studies. For each assay, one pellet of dried inactivated syt.427 or control interfering RNA yeast that had been prepared as described above was resuspended in 300 ul of water. A toothpick was used to insert holes into 15 mL of standard fly food medium, and the yeast solution, which was mixed with McCormick’s red food dye, was pipetted into the holes. In each biological replicate assay, the control or syt.427 yeast-food mixture was fed to 20 first instar larvae that were placed in the vial of food and reared to adulthood. Consumption of the yeast was confirmed through observation of red food dye in the larval guts throughout the experimental period. These assays were performed under ambient laboratory illumination (12 hr light/12 hr dark) at 22°C. The number of adults that emerged from each tube was observed and recorded as a measurement of survival. The Fischer’s exact test was used to evaluate data combined from three biological replicate experiments.

*Daphnia*: *Daphnia magna* and *Daphnia pulex* were purchased from Carolina Biologicals (Burlington, NC). 10 adults of each species were reared on either control or syt.427 yeast in each of three biological replicate assays. These studies were conducted under ambient laboratory illumination (12 hr light/12 hr dark) at 22°C in COMBO medium containing 0.0001% sodium selenium [30]. A single yeast pellet (control or syt.427) was dissolved in 50 mL of distilled water, and 10 mL of this solution was fed to the animals every day for five days. Survival of the *Daphnia* was assessed daily throughout a 10 day trial period. Survival data from three replicate experiments were combined and assessed with the Fischer’s exact test.

## Results and discussion

### Larval consumption of interfering RNA targeting *Aae syt* induces high levels of mortality

siRNA #427, which corresponds to a target sequence in the third exon of *Aae syt* (S1 Table and S1 Fig), was identified in a larval siRNA soaking screen conducted on L1 larvae in which it induced 32.5±1.3% larval death (Fig 1A; P = 0.00008 vs. control siRNA treatment). Based on the soaking results for siRNA #427 (Fig 1A), it was therefore hypothesized that yeast interfering RNA larvicides targeting the same sequence would induce high levels of larval mortality. To test this, *S*. *cerevisiae* was transformed with a non-integrating multi-copy yeast shuttle plasmid from which shRNA corresponding to the #427 target sequence was placed under control of a constitutive promoter. #427 yeast, as well as control yeast expressing shRNA with no known target in mosquitoes, was heat-killed and prepared into an inactivated dry tablet formulation. Although larvae fed with control yeast interfering RNA tablets survived (Fig 1B), yeast in which #427 shRNA had been expressed induced 91.5±0.8% mortality (Fig 1B; P = 1.87X10^-18^ vs. control yeast interfering RNA treatment).

**Fig 2:**
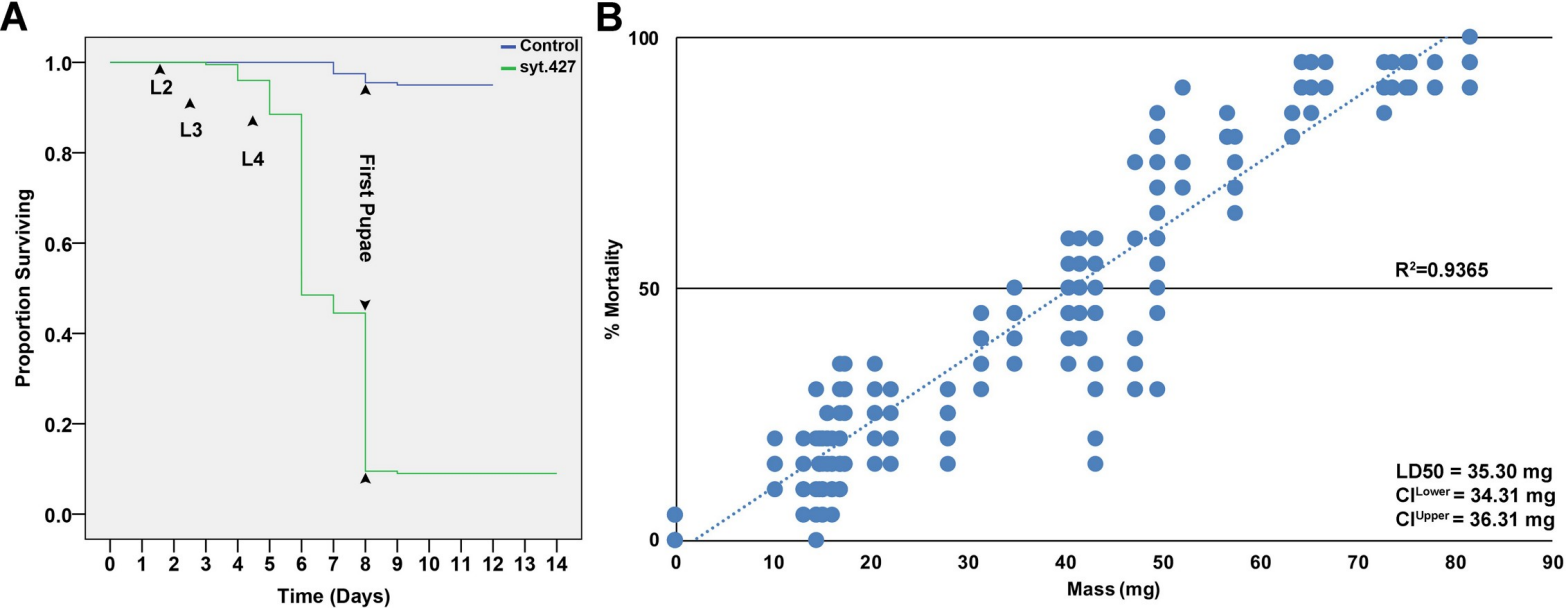
Assessment of yeast interfering RNA larvicide syt.427 activity in *A*. *aegypti*. A. Consumption of inactivated dried yeast interfering RNA syt.427 larvicide tablets induced larval mortality in the L4 larval or pupal stages (days 4–8; compare to larvae fed with control yeast interfering RNA that survived and pupariated). B. Dose-response curves depicting the mass of syt.427 vs. the percentage of *A*. *aegypti* larval mortality are shown. LD_50_ values for syt.427 with upper and lower confidence limits (CL) are indicated. Regression analysis demonstrated that the dose-response data were linearly correlated (R^2^ = 0.9365). Additional details regarding calculation of lethal doses are provided in the methods section.

As discussed previously [7], when semi-field studies are planned, it is useful to prepare stably transformed *S*. *cerevisiae* strains, which eliminates the use of plasmids with antibiotic resistance markers and mitigates the potential for horizontal transfer of shRNA expression cassettes. Given the high rates of larval mortality observed in larvae fed with yeast transiently transformed with the #427 shRNA expression plasmid (Fig 1B), *S*. *cerevisiae* that were stably transformed with the #427 shRNA expression construct were generated. Dry inactivated yeast interfering RNA tablets were prepared from the stably transformed yeast strain, hereafter referred to as larvicide syt.427. A comparable stable transformant strain induced to express control shRNA with no known target in mosquitoes [7] was used as a control for all experiments in this investigation and is hereafter referred to as the control. Larval consumption of syt.427 yeast resulted in 92.7±1.0% larval mortality (Fig 1C; P = 1.10X10^-18^ vs. control yeast interfering RNA treatment) in laboratory trials conducted in containers bearing 20 *A*. *aegypti* larvae (LD_50_ = 35.3 mg; Fig 2B). Larvae treated with syt.427 beginning in L1 died in L4 or as early pupae (Fig 2A; similar results were obtained for the plasmid-based syt.427 yeast strain). Similarly, 90.1±1% mortality was observed when syt.427 yeast was fed to 200 larvae reared in 1 L containers (P = 2.89X10^-26^ vs. control-treated larvae, for which 4.6±1% mortality was observed). These data suggest that syt.427 yeast larvicides, like other *A*. *aegypti* yeast interfering RNA larvicides generated in previous studies [7], can effectively kill larvae reared at higher densities.

The adult survivors of syt.427 treatment (~9% of treat larvae) were further assessed. No significant differences were observed in the fertility (P = 0.19), fecundity (P = 0.072), or longevity of females (P = 0.41) that had been reared on syt.427, control interfering RNA yeast, or *CEN*.*PK* yeast lacking the shRNA expression construct (S2 Table). Given that dead larvae were seldom observed in syt.427-treated containers, it seems likely that survivors of syt.427 treatment may be eating other dead larvae in the container instead of syt.427 yeast. In support of this idea, 100% of larvae treated with syt.427 died when the larvae were raised individually (Fig 1D; P = 1.09X 10^−47^ vs. control), and the remains of these larvae could be observed for several days.

### Yeast interfering RNA larvicide syt.427 silences *Aae syt* expression in the larval brain and disrupts neural synapses

**Fig 3:**
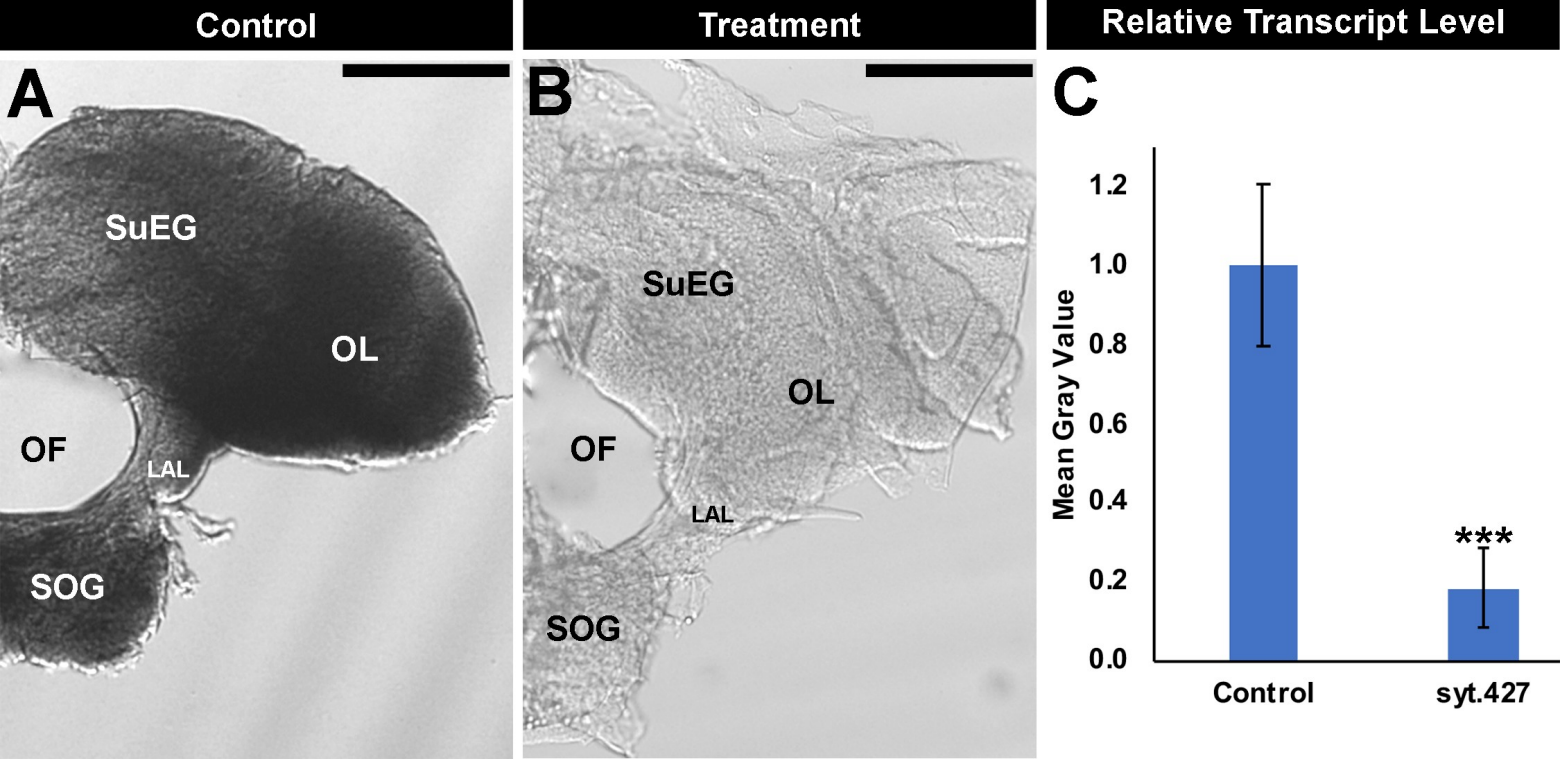
Silencing of *Aae syt* expression in the larval brain following syt.427 consumption. The high levels of *Aae syt* expression detected throughout the *A*. *aegypti* L4 brain (control-treated larvae shown in A) were significantly reduced in larvae fed with dried inactivated yeast interfering RNA syt.427 tablets (B). Mean gray value results from three biological replicate experiments were compiled (C; n = 60 for syt.427-treated brains; n = 55 for control-treated brains), and these data were evaluated with a paired t-test (C). *** = P<0.001 when compared with control-fed larvae; data are represented as average mean gray values, and error bars denote SEM. Representative brains from these experiments are oriented dorsal upward in this figure. **LAL:** larval antennal lobe; **OF:** olfactory foramen; **OL:** optic lobe; **SOG:** sub-esophageal ganglion; **SuEG:** supra-esophageal ganglion. Scale Bar = 100 μm.

As discussed above, yeast interfering RNA larvicide syt.427 contains shRNA that corresponds to a target sequence in the *Aae syt* gene (S1 Table and S1 Fig). The function of *Drosophila melanogaster synaptotagmin 1 (syt1)*, ortholog of the *Aae syt* gene, has been studied in detail in *Drosophila* and is known to be conserved in mammals [31–33]. The release of neurotransmitters following an action potential requires rapid fusion of synaptic vesicles in response to Ca^2+^ influx, a process controlled by the evolutionarily conserved SNARE complex that regulates fusion of presynaptic vesicles with the neuronal plasma membrane (reviewed by [32]). Syt1, the Ca^2+^ sensor for vesicle fusion, controls the precise timing and release of neurotransmitters from presynaptic neurons that is critical for synaptic transmission and normal brain function [9, 32–36]. Syt1 is expressed broadly in the *D*. *melanogaster* nervous system, in which it maintains presynaptic localization throughout development [37]. Similar to *D*. *melanogaster*, in which Syt1 expression is detected throughout the late larval brain [38], *Aae syt* is expressed broadly in the larval brain of early fourth instar *A*. *aegypti* larvae (Fig 3A). Based on this expression pattern, and given that the presynaptic neural functions of Syt1 are well-conserved in invertebrate and vertebrate organisms [12], it was hypothesized that loss of *Aae syt1* would impact presynaptic neural function during larval development.

**Fig 4:**
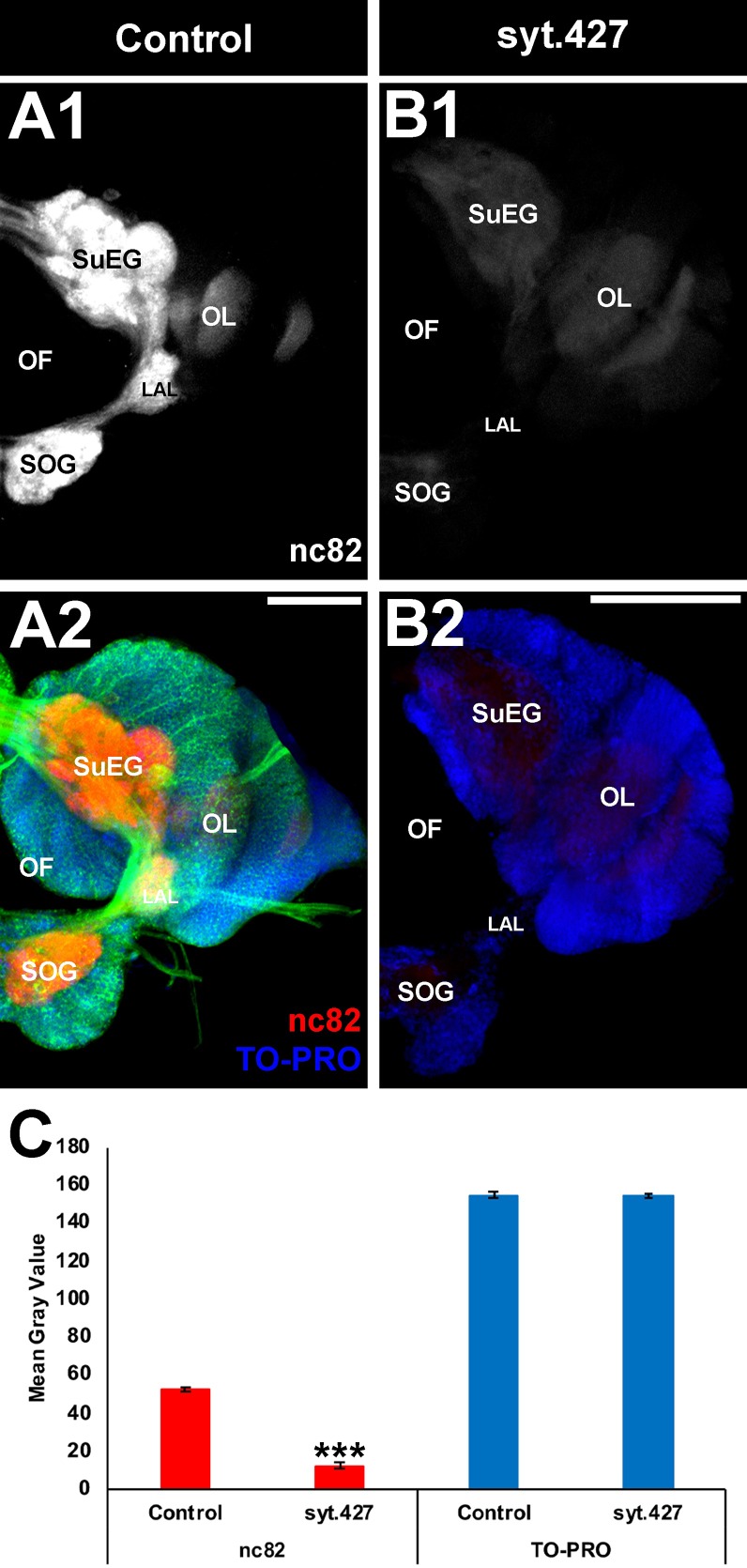
Neural defects observed in the L4 brains of larvae fed with yeast interfering RNA larvicide syt.427. Stage L4 larval brains were labeled with mAbnc82 (white in A1, B1; red in A2, B2), which labels synaptic active zones. nc82 levels were significantly reduced (C) in the synaptic neuropil of L4 larvae that had consumed syt.427 (B1, B2; compare to white staining of control brain in A1/red staining in A2), but levels of TO-PRO, which marks nuclei, were not significantly different (C) between syt.427- (B2) and control-treated (A2) larvae. Data compiled from three biological replicate experiments containing a total of 80 syt.427-treated and 49 control-treated larvae are shown and were evaluated with a paired t-test (C). Data are represented as average mean grey values, and error bars denote SEM (C). *** = P<0.001 when compared with control-fed larvae. Representative brains from these experiments are oriented dorsal upward in this figure. **LAL:** larval antennal lobe; **OF:** olfactory foramen; **OL:** optic lobe; **SOG:** sub-esophageal ganglion; **SuEG:** supra-esophageal ganglion. Scale Bar = 100 μm.

The effects of yeast interfering RNA larvicide syt.427 were assessed in the *A*. *aegypti* nervous system during early L4, just prior to the time at which these larvae die (Fig 2A). Quantification of transcript levels in the brains of L4 larvae fed with control (Fig 3A) vs. syt.427 (Fig 3B) yeast interfering RNA larvicide confirmed that syt.427 treatment results in significant silencing of *Aae syt* expression (Fig 3C; 79.1±11.6% reduction of *syt1* transcripts; P = 8.54X10^-50^). nc82 antibody staining, which reveals expression of Bruchpilot (Brp), a marker of presynaptic active zones [27], was assessed in syt.427-treated larvae (Fig 4). Although levels of the nuclear marker TO-PRO were not significantly different in syt.427-treated vs. control larvae (Fig 4A2, 4B2 and 4C), nc82 levels were significantly reduced in the brains of L4 larvae that had consumed syt.427 (Fig 4; 77% reduction in levels with respect to control-treated brains; P = 2.53X10^-39^). These results indicate that although neural densities in the larval brain are not altered by consumption of syt.427 larvicide, the resulting silencing of *Aae syt* impacts presynaptic neural activity. This disruption of presynaptic activity in the larval nervous system correlated with the timing of larval death (Fig 2A) and is likely a primary cause of mortality in *A*. *aegypti* larvae that consume syt.427. The results of these experiments indicate that the mode of action for yeast interfering RNA larvicide syt.427 is silencing of the *Aae syt* gene, which results in defective presynaptic active zones.

### Simulated-field and semi-field evaluation of syt.427 activity

**Fig 5:**
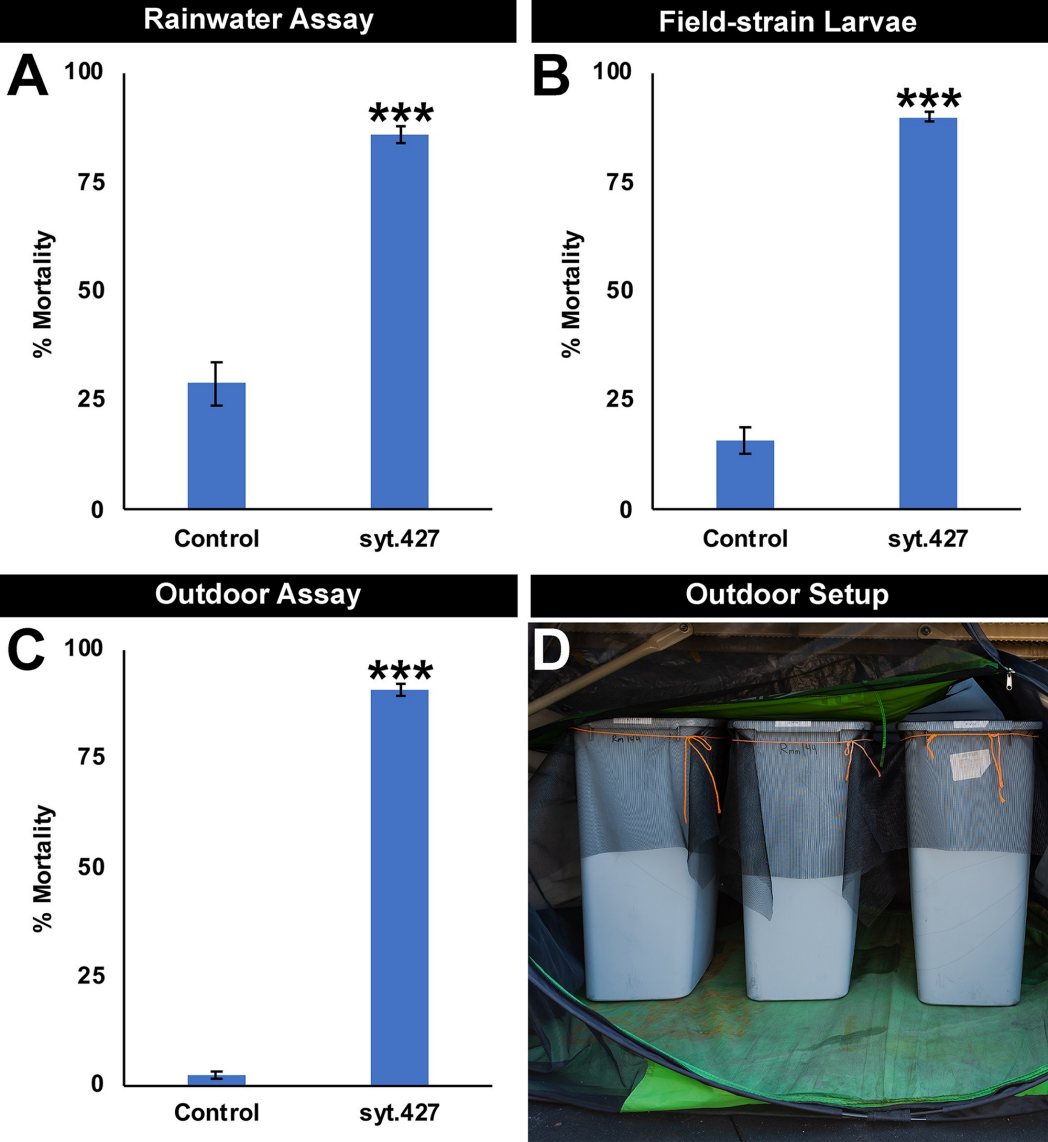
Simulated-field and semi-field evaluation of syt.427 activity. Activity of syt.427 was confirmed in assays conducted with rainwater (A; data shown are from replicate experiments that included a total of 120 larvae per treatment) and using larvae from a field strain of *A*. *aegypti* mosquitoes generated from eggs collected in Trinidad (B; data shown were combined from replicate experiments that included a total of 240 larvae per treatment). High levels of larval mortality were induced by syt.427 in semi-field trials (C) conducted in a contained outdoor roof top laboratory (D). The semi-field data shown (C) were compiled from multiple biological replicate experiments conducted on a total of 180 larvae/treatment. Data are represented as mean percentage larval mortality and were evaluated with t-tests; error bars denote SEM. ***P<0.001 when compared with control-fed larvae.

In preparation for future field studies, syt.427 activity was evaluated under conditions that more closely simulate field conditions. First, syt.427 activity was confirmed in insectary experiments conducted using rainwater rather than sterile distilled water (Fig 5A; P = 0.00025). These results indicated that yeast interfering RNA larvicide activity is not dependent on the use of sterile water, an important criterion for larvicides that will succeed in the field. Next, syt.427 activity was confirmed in insectary experiments that were conducted using larvae collected from a newly-generated field strain of *A*. *aegypti* mosquitoes recently established from eggs collected using ovitraps in Trinidad, Trinidad and Tobago (5B; P = 5.99X10^-11^). These results suggest that the yeast tablet feeding behavior of Trinidad field strain mosquitoes does not differ substantially from lab strains. The results also indicate that the target site of syt.427 is conserved in different strains of *A*. *aegypti* mosquitoes, which is to be expected given its conservation in different species of disease vector mosquitoes (S1 Table and S1 Fig).

In further preparation for future field assessment of syt.427 activity, semi-field testing of yeast interfering RNA larvicide syt.427 was pursued in an outdoor roof top laboratory. In these assays, container sizes were increased to 30 L containers bearing 26 L of water, a size that better approximates the size of the most productive *A*. *aegypti* larval breeding sites in the tropics [39]. Although little death was observed in containers treated with control yeast, 90.6±1.3% larval death was observed in syt.427-treated containers (Fig 5C; p = 6.85X10^-10^). These results suggest that yeast interfering RNA larvicide syt.427 activity is retained during exposure to outdoor conditions and temperatures that ranged from 13.5°C to 42.0°C during the testing period.

### Yeast interfering RNA larvicide syt.427 functions as a broad-range mosquito larvicide

**Fig 6:**
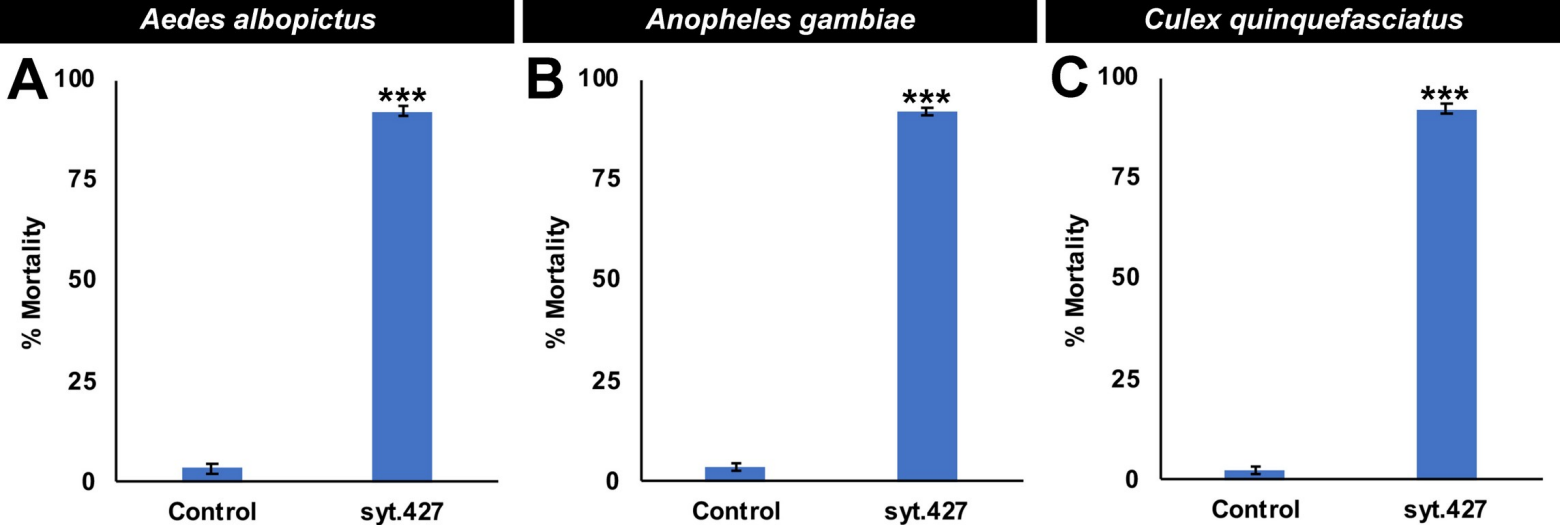
syt.427 is a broad-range mosquito larvicide. Consumption of syt.427 inactivated yeast interfering RNA larvicide tablets induces high levels of mortality in *A*. *albopictus* (A), *A*. *gambiae* (B), and *C*. *quinquefasciatus* (C) larvae. Results were compiled from multiple biological replicates on a total of 180 *A*. *albopictus* (A) 240 *A*. *gambiae* (B), and 240 *C*. *quinquefasciatus* (C) larvae per treatment. Data are represented as mean larval mortality, and error bars denote SEM. Data were evaluated with a t-test. *** = P<0.001 when compared with control-fed larvae.

The target site of yeast interfering RNA larvicide syt.427 is conserved in multiple species of *Anopheles* malaria vector mosquitoes, as well as *A*. *albopictus* and *C*. *quinquefasciatus*, but not in insects, humans, or other non-target organisms (S1 Table and S1 Fig). Recent studies have demonstrated that yeast interfering RNA larvicides can be used to silence *A*. *gambiae* larval genes [8], and it was therefore hypothesized that yeast interfering RNA larvicide syt.427 would induce significant larval mortality in this species. As predicted, yeast interfering RNA larvicide syt.427 induced 92.0±1.0% larval mortality in *A*. *gambiae* (Fig 6B; P = 1.18X10^-13^ vs. control yeast interfering RNA treatment). In addition to *A*. *gambiae*, the syt.427 target site is conserved in multiple other species of *Anopheles* mosquitoes (S1 Table), suggesting that it could potentially be used for control of multiple malaria vectors. To this end, the WHO recommends that when it is employed as a supplement to insecticide treated nets and indoor residual spraying, larviciding can be applied for *Anopheles* control in urban settings where vector breeding sites are few, fixed, and findable [40]. Yeast interfering RNA larvicides targeting malaria vector mosquitoes could potentially address multiple high-priority needs, including prevention of residual transmission, targeting of immature mosquitoes in outdoor aquatic habitats, the control of outdoor biting mosquitoes, protection of outdoor workers, the prevention of insecticide resistance, and the need to control multiple species of mosquitoes that vector malaria parasites [8, 41].

**Fig 7:**
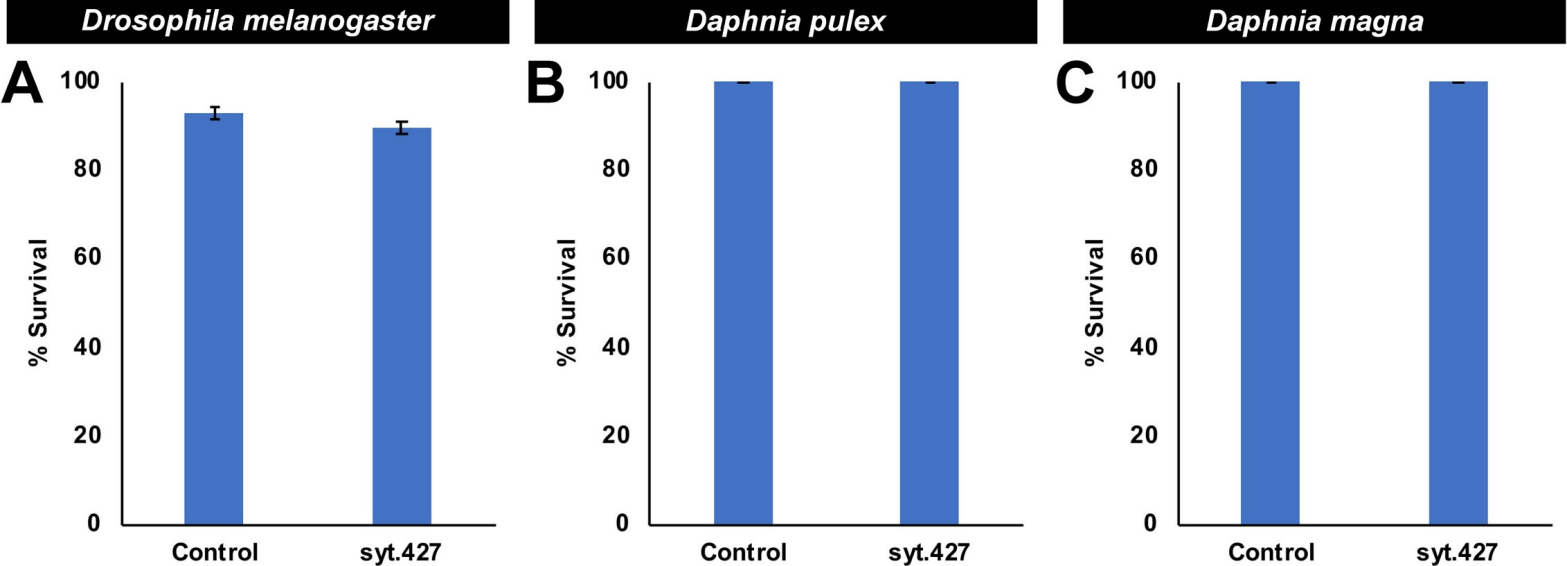
Three non-target arthropods survive syt.427 treatment. Consumption of syt.427 yeast by *D*. *melanogaster* larvae (A) did not impact survival through adult emergence (a total of 60 larvae/treatment were assessed in three biological replicate experiments). Consumption of syt.427 by *D*. *pulex* (B) or *D*. *magna* (C) did not impact adult survival (a total of 30 animals/treatment were evaluated in three biological replicate experiments conducted on each species). Survival data compiled from three replicate experiments and analyzed with the Fisher’s exact test revealed no significant differences in the survival of syt.427- and control-treated organisms. Graphs display mean percentages of survival.

Although yeast interfering RNA larvicides had not yet been assessed in *A*. *albopictus* or in *C*. *quinquefasciatus*, it was hypothesized, based on conservation of the syt.427 target site in these species (S1 Table and S1 Fig) and the observed activity of this larvicide in *A*. *aegypti* (Figs 1, 2, 3, 4 and 5) and *A*. *gambiae* (Fig 6B), that syt.427 tablets would also induce mortality in both species. Yeast interfering RNA larvicide syt.427 induced significant larval mortality in *A*. *albopictus* (Fig 6A), in which 92.0±1.0% mortality was observed in fourth instar larvae (P = 3.50X10^-11^ vs. control yeast interfering RNA treatment). Likewise, significant larvicidal activity was observed in *C*. *quinquefasciatus* (Fig 6C), with 92.0±1.0% of larvae dying in the fourth instar (P = 1.53X10^-11^ vs. control yeast interfering RNA treatment). Although syt.427 kills a variety of mosquito species, it has no larvicidal activity in *D*. *melanogaster*, a dipteran insect in which the syt.427 target site was not identified (S1 Table and Fig 7A; no significant difference in control vs. syt.427 larval survival). Similarly, *D*. *pulex* (Fig 7B) and *D*. *magna* (Fig 7C), two distantly related aquatic arthropods that are often utilized in U.S. Environmental Protection Agency (EPA) toxicity assays [42], lack the syt.427 target site (S1 Table) and survived treatment with syt.427 yeast (Fig 7B and 7C; no significant difference in control vs. syt.427 treated larval survival were found). Combined, these results demonstrate that syt.427 may represent a new tool for the biorational control of multiple disease vector mosquitoes.

In general RNA-based products appear to have an overwhelmingly desirable safety profile, particularly when compared to conventional pesticides [43], as evidenced by the survival of non-target organisms treated with syt.427 (Fig 7). However, it will of course be critical to perform toxicology tests on additional species using commercially-ready formulations, particularly given that it is difficult to predict on the basis of sequence alone whether any given interfering RNA molecule could have non-target impacts [44]. The United States EPA recently approved an RNAi-based genetically modified organism as an agricultural pesticide tool [45], and it is likely that additional registry applications will soon follow. Pursuit of field testing for yeast interfering RNA larvicides in the United States would be an important step toward future EPA registry applications. However, the use of genetically modified yeast, even if it is heat-inactivated, will need to be approved in each country of intended use throughout the world. This will be challenging, as some countries still lack a regulatory body equivalent to the EPA to review such new technologies. Despite these challenges, pursuit of further toxicology testing, field testing, and EPA registry of syt.427 could increase the likelihood of gaining approval for its use in other countries.

An added advantage of gaining regulatory approvals for syt.427 is that this larvicide, unlike many other RNAi pesticide technologies, can be used for treatment of multiple disease vector mosquitoes (Fig 6), including both *A*. *aegypti* and *A*. *albopictus*. *A*. *albopictus*, like *A*. *aegypti*, vectors dengue, Zika, and chikungunya viruses, and the spatial distributions of *A*. *aegypti* and *A*. *albopictus* frequently overlap [46]. Interspecific encounters between the two *Aedes* species are diverse and range from wide-spread competitive displacements (such as the displacement of *A*. *aegypti* by *A*. *albopictus* in the southeastern United States) to local habitat segregation, as well as instances in which no apparent effects are observed [47]. Despite the complexity of these interactions, the ability to control both species with the same larvicidal agent is advantageous, particularly given that the two species may lay eggs in the same container breeding sites, the surveying of which is complicated by the lack of distinct morphological features in the larval stages. The ready-to-use syt.427 yeast interfering RNA larvicide tablets assessed in this investigation may therefore represent a new biorational approach for control of both species that can be seamlessly integrated with existing strategies for control of these disease vector mosquitoes in container breeding sites. The residual activity of these tablets (presently ~10 days; [7]) could be improved through the development of long-lasting formulations [11], and this is a priority goal.

Given the recent spread of West Nile virus across the continental United States, the existing global disease burden of lymphatic filariasis [3], as well as reported resistance to existing larvicides [48], it is also useful that syt.427 has larvicidal activity in *C*. *quinquefasciatus* (Fig 6C). Larvicides are often used to treat stormwater catch basins, common sources of permanent or semi-permanent standing water in urban and suburban areas that are known to be important breeding sites for *Culex* mosquitoes [49–51]. Interestingly, Arana-Guardia et al. [52] recently collected both *C*. *quinquefasciatus* and *A*. *aegypti* from catch basins for stormwater in Merida City, Mexico, concluding that these catch basins are productive sources of both species of larvae during both the wet and dry seasons. Their results [52] suggest that use of syt.427 larvicides in catch basins could permit bioratonal control of both *A*. *aegypti* and *C*. *quinquefasciatus*. It may therefore be useful to develop formulations of yeast interfering RNA larvicides that can persist in catch basins. Nasci et al. [4] recently evaluated the effectiveness of a variety of different larvicides for controlling *C*. *pipiens* larvae in urban stormwater catch basins in Illinois, USA. Although the results of their studies indicated that all the products tested provided measurable levels of control, the authors concluded that monthly re-treatments with granular formulations may be more cost-effective than using fewer applications of extended-duration larvicides in catch basins located in areas that were difficult to control. The authors speculated that briquette and tablet formulations may be prone to being buried in sediment or flushed out of the catch basin, but that granular formulations may be more readily dispersed and less prone to becoming flushed away or buried. It will be interesting to develop additional formulations of yeast interfering RNA larvicides, including granular vs. tablet and shorter vs. extended release formulations, and to determine which are most useful for treatment of catch basins. Additionally, although the present yeast interfering RNA tablet formulation sinks to the bottom of containers, based on the findings of Nasci et al. [4], it may be useful to develop more buoyant formulations of yeast interfering RNA larvicides that are more readily dispersed. Likewise, such formulations may be more appropriate for treatment of large barrels and drums that are often used to store drinking water in the tropics, and which are some of the most productive *Aedes* breeding containers [39].

### Conclusions and future directions

The results of this investigation demonstrate that syt.427, a yeast interfering RNA larvicide with a target site conserved in *A*. *aegypti*, *A*. *albopictus*, *C*. *quinquefasciatus*, as well as a variety of *Anopheles spp*. mosquitoes, but which is not conserved in humans or other non-target organisms (S1 Table and S1 Fig), may offer a new biorational means of controlling a variety of disease vector mosquito species. Our studies demonstrate that *S*. *cerevisiase* may be an excellent system for production and delivery of interfering RNA molecules to a variety of different species of mosquito larvae. Both our previous work and the results of the present investigation demonstrated that interfering RNA larvicides are effective in laboratory trials conducted against *A*. *aegypti* (Figs 1 and 2, and reference [7]) and *A*. *gambiae* (Fig 6B and reference [8]) mosquitoes. The present study extends the potential use of this technology to both *A*. *albopictus* and *C*. *quinquefasciatus* mosquitoes, describing characterization of a single yeast interfering RNA larvicide that can target all of these mosquitoes (Fig 6) and potentially a number of additional malaria vector mosquito species (S1 Table and S1 Fig). Expression of dsRNA in *S*. *cerevisiae* was recently used to target vital genes in the agricultural pest *Drosophila suzukii* [53], providing further evidence that yeast interfering RNA technology could be used to target any number of yeast-eating insect pests. Moreover, the development of yeast interfering RNA larvicides, a novel class of insecticides, will help to combat resistance to existing mosquito larvicides [54–60]. By building an arsenal of different yeast interfering RNA larvicide strains, we are beginning to combat resistance that could develop due to a mutation in any one shRNA target site.

In this investigation, our confirmation of yeast interfering RNA larvicide activity in laboratory trials that better simulate field conditions, as well as in semi-field trials (Fig 5), provide support that this technology could be implemented successfully in the field. However, in preparation for successful field trials, it will likely be critical to develop a variety of different formulations, including tablets, granules, and briquettes, as well as formulations with varied buoyancies. This would permit the most effective treatment of different species of disease vector mosquitoes living in a variety of different habitats. The identification of encapsulating agents that promote yeast stability in various environmental conditions, both prior to and during its use, will be important, particularly for the treatment of mosquitoes that may breed in water that contains higher amounts of organic materials than the roof-runoff rainwater evaluated in these studies (Fig 5A). These encapsulated formulations could also facilitate controlled and extended release of yeast interfering RNA larvicides, promoting increased residual activity [11]. We anticipate that a variety of different ready-to-use inactivated formulations of biorational yeast interfering RNA larvicides could seamlessly integrate, with minimal educational and training campaigns, into existing mosquito control programs. In preparation for successful commercialization of this intervention, it will also be critical to investigate the potential for culturing yeast interfering RNA strains at industrial-sized scale and to assess how the strains and their growth conditions can be further optimized. The genetic tractability of *S*. *cerevisiae* and extensive history of using this microbe in both the food and pharmaceutical industry will surely benefit the development of this microbe as a production and delivery system for interfering RNA pesticides [11].

## Supporting information

S1 Table Evaluation of syt.427 target site conservation. The 21 bp sequence targeted by syt.427 was used as a query sequence against all *Aedes*, *Anopheles*, and *Culex* genomes in Vectorbase. Mosquito species bearing a perfectly conserved target sequence are listed along with the corresponding gene identification numbers (if known) or scaffold (s) locations of the conserved target sites. The 21 bp target sequence was also used as an input for NCBI blastn searches conducted against the indicated taxonomic groups, for which corresponding taxonomic identification numbers (TaxIDs) are listed. As of September 2018, searches against all sequences in the blast database did not uncover any perfect matches outside of disease vector mosquitoes.
(PDF)

S2 Table Assessment of adults that survive syt.427 treatment as larvae. No significant differences were observed in the fecundity (number of eggs laid), fertility (percentage of eggs hatched), or longevity of surviving adult females that had been reared on yeast tablets prepared from the syt.427, control interfering RNA, or *CEN*.*PK* (lacks an shRNA expression construct) yeast strains (P>0.05). Please see text for additional experimental details. SEM = Standard error of the mean.
(PDF)

S1 Fig Conservation of the syt.427 target site in mosquito *syt* genes. A. The location of the syt.427 target site in exon 3 (E3) of *Aae syt* (AAEL000704) is marked by an arrow. Exon/intron structure information for this gene was exported from Vectorbase [61]; the exons are shown as boxes, with filled boxes denoting coding regions. B. The Clustal Omega [62] alignment of *Aae syt* exon 3 with corresponding homologous sequences in other mosquito *syt* orthologs is shown (see S1 Table for corresponding species information). In some orthologs, the region of homologous sequence corresponds to exon 2 (E2) as indicated. The conserved syt.427 target site is highlighted in green.
(PDF)
