# Supplementary material for: Characterization of a yeast interfering RNA larvicide with a target site conserved in the synaptotagmin gene of multiple disease vector mosquitoes
Source: PLoS Negl Trop Dis. 2019 May 20;13(5):e0007422. doi: 10.1371/journal.pntd.0007422 (PMC6544322; doi:10.1371/journal.pntd.0007422)
Supplement: S1 Table — The 21 bp sequence targeted by syt.427 was used as a query sequence against all Aedes, Anopheles, and Culex genomes in Vectorbase. Mosquito species bearing a perfectly conserved target sequence are listed along with the corresponding gene identification numbers (if known) or scaffold (s) locations of the conserved target sites. The 21 bp target sequence was also used as an input for NCBI blastn searches conducted against the indicated taxonomic groups, for which corresponding taxonomic identification numbers (TaxIDs) are listed. As of September 2018, searches against all sequences in the blast database did not uncover any perfect matches outside of disease vector mosquitoes. (PDF) [file pntd.0007422.s001.pdf]

**Mosquito Species or Taxonomic Group/Taxonomic ID**	**Identical Match?**	**Match Location**
Aedes aegypti	Yes	AAEL000704
Aedes albopictus	Yes	XM_019705510.1
Anopheles albimanus	Yes	KB672397A (s)
Anopheles atroparvus	Yes	AATE010001
Anopheles arabiensis	Yes	KB704374 (s)
Anopheles culicifacies	Yes	ACUA027427
Anopheles dirus	Yes	ADIR005979
Anopheles epiroticus	Yes	AEPI006998
Anopheles farauti	Yes	AFAF002055
Anopheles funestes	Yes	KB668892 (s)
Anopheles gambiae	Yes	AGAP007942
Anopheles merus	Yes	AMEM008390
Anopheles minimus	Yes	KB663733 (s)
Anopheles quadrimaculatus	Yes	AQUA000722
Anopheles sinensis	Yes	ASIC007305
Anopheles stephensi	Yes	ASTEI07666
Culex quinquefasciatus	Yes	CPIJ004781
Amphibians/8292	No	N/A
Birds/8782	No	N/A
Fish/7898	No	N/A
Fungi/4751	No	N/A
Human/9606	No	N/A
Mammals/40674	No	N/A
Plants/3193	No	N/A
Reptiles/8504	No	N/A
