# Supplementary material for: Characterization of a yeast interfering RNA larvicide with a target site conserved in the synaptotagmin gene of multiple disease vector mosquitoes
Source: PLoS Negl Trop Dis. 2019 May 20;13(5):e0007422. doi: 10.1371/journal.pntd.0007422 (PMC6544322; doi:10.1371/journal.pntd.0007422)
Supplement: S2 Table — No significant differences were observed in the fecundity (number of eggs laid), fertility (percentage of eggs hatched), or longevity of surviving adult females that had been reared on yeast tablets prepared from the syt.427, control interfering RNA, or CEN.PK (lacks an shRNA expression construct) yeast strains (P>0.05). Please see text for additional experimental details. SEM = Standard error of the mean. (PDF) [file pntd.0007422.s002.pdf]

S2 Table. Assessment of adults that survive syt.427 treatment as larvae.

	Fecundity (# eggs)		Fertility (% hatched)		Survival (%)	
	Mean	SEM	Mean	SEM	Mean	SEM
CEN.PK	89	3	85	2	86	6
**control**	99	3	91	2	75	7
**syt.427**	93	3	88	3	83	9
