# Supplementary material for: Characterization of a yeast interfering RNA larvicide with a target site conserved in the synaptotagmin gene of multiple disease vector mosquitoes
Source: PLoS Negl Trop Dis. 2019 May 20;13(5):e0007422. doi: 10.1371/journal.pntd.0007422 (PMC6544322; doi:10.1371/journal.pntd.0007422)
Supplement: S1 Fig — A. The location of the syt.427 target site in exon 3 (E3) of Aae syt (AAEL000704) is marked by an arrow. Exon/intron structure information for this gene was exported from Vectorbase [61]; the exons are shown as boxes, with filled boxes denoting coding regions. B. The Clustal Omega [62] alignment of Aae syt exon 3 with corresponding homologous sequences in other mosquito syt orthologs is shown (see S1 Table for corresponding species information). In some orthologs, the region of homologous sequence corresponds to exon 2 (E2) as indicated. The conserved syt.427 target site is highlighted in green. (PDF) [file pntd.0007422.s003.pdf]

A

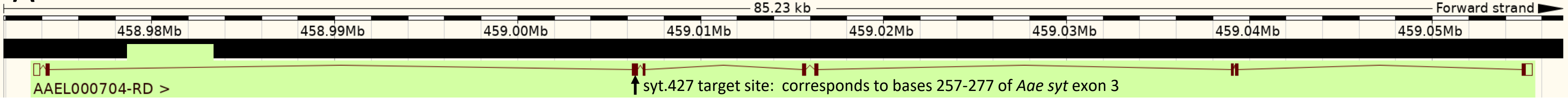

B

```
CPIJ004781:E2       AAGAAAATGACGAGTACTCGAGTACCGCGGCGCCAACGAAGGGCGAGGCGGAAAAGATCA    60
AAEL000704:E3       ACGAAAATGACGAGCTTGGAAGCACCGCGTCGCCAACGAAAGGCGAGGCGGAGAAGATTG    60
XM_019705510.1      ACGAAAATGACGAGCTTGGAAGCACCGCTTCGCCAACGAAAGGCGAAGCGGAGAAGATTG    60
AATE010001:E3       ---AAGAGAATGACGATTTCAGCACAGCGTTGCCAACCAAGGGCGAGGCGGAAAAGCTGG    57
ASIC007305:E2       ---AAGAGAATGAGGACTTTAGCACAGCGTTGCCGACCAAGGGCGAGGCGGAAAAGCTGG    57
AEPI006998:E2       ---AAGAAAACGAAGATTTTAGCACGATCTCCCCCACCAAAGGAGAGGCGGAAAAGCTGG    57
ACUA027427:E2       ---AAGAGAATGAAGATTTCAGCACAGCTTCGCCCACCAAAGGGGAGGCGGAAAAGTTGG    57
AGAP007942:E3       ---AGGAAAATGAGGATTTCAGCACGGCAACGCCAACCAAAGGCGAGGCGGAAAAGCTGG    57
AMEM008390:E3       ---AGGAAAATGAGGATTTCAGCACGGCAACGCCAACCAAAGGCGAGGCGGAAAAGCTGG    57
ASTEI07666:E2       ---AGGAGAACGAAGATTTCAGCACAGCATCCCCGACCAAGGGAGACGCGGAAAAGCTGG    57
ADIR005979:E3       ---AAGAGAATGAGGATTTCAGCACAGCCTCGCCGACCAAAGGGGAAGCGGAAAAACTGG    57
AFAF002055:E3       ---AAGAGAATGAGGATTTCAGCACAGCATCGCCAACCAAGGGAGAGGCCGAAAAGCTGG    57
                       *  *  * **       ** **       ** ** ** ** ** ** ** **  *

CPIJ004781:E2       GCGAGAAGGCGGCGGAAATCACCGAGGCCCTGGCACACGAGATGGGCATCCCGACGTGGG    120
AAEL000704:E3       GCGAGAAGGCGGCGGAAATCACCGAGGCACTAGCACACGAAATGGGCATCCCAACGTGGG    120
XM_019705510.1      GTGACAAAGCGGCAGAGATCACCGAGGCCCTGGCAAGCGAAATGGGCATACCAACGTGGG    120
AATE010001:E3       GAGACAAGGCGGCCGAGATGACCGAGGCACTGGCGCACGAGATGGGGATTCCGACGTGGG    117
ASIC007305:E2       GAGACAAGGCGGCCGAGATGACCGAGGCCCTGGCGCACGAGATGGGCATTCCAACGTGGG    117
AEPI006998:E2       GAGACAAGGCGGCTGAGATGACCGAGGCGTTAGCTCATGAGATGGGCATTCCAACCTGGG    117
ACUA027427:E2       GCGACAAGGCGGCCGAGATGACGGAGGCACTGGCGCACGAGATGGGCATACCGACCTGGG    117
AGAP007942:E3       GAGACAAGGCGGCCGAGATGACGGAGGCGTTAGCCCACGAGATGGGCATCCCGACGTGGG    117
AMEM008390:E3       GAGACAAGGCGGCCGAGATGACGGAGGCGTTAGCCCACGAGATGGGCATTCCGACGTGGG    117
ASTEI07666:E2       GCGATAAGGCGGCCGAGATGACCGAGGCGCTAGCGCACGAGATGGGCATTCCGACGTGGG    117
ADIR005979:E3       GCGATAAGGCGGCCGAGATGACCGAAGCGCTAGCTCACGAAATGGGCATCCCAACGTGGG    117
AFAF002055:E3       GCGATAAGGCGGCCGAGATGACCGAAGCGTTAGCGCACGAAATGGGAATCCCAACATGGG    117
                    * ** ** ***** ** ** ** ** **  * **    ** ***** ** ** ** ****

CPIJ004781:E2       GCCTCGTGTCCATCATCATCGTCGTCTGCCTGATCATCCTCGGCATCTGCGGCTTCTGCA    180
AAEL000704:E3       GCCTAGTGTCCATCATAATCGTGGTCGTCCTGATCATCCTCGGCATCTGTGGCTTCTGCA    180
XM_019705510.1      GCCTGGTGTCGATCATAATCGTGGTCCTCCTGATCATCCTCGGCATCTGTGGCTTCTGCA    180
AATE010001:E3       GACTCGTGTCCATTATCATCTTCATAGTACTGATTATCCTGGGCATATGCGGCTTCTGCA    177
ASIC007305:E2       GTCTTGTGTCCATCATCATCTTCGTAGTACTGATTATCCTGGGCATATGCGGTTTCTGCA    177
AEPI006998:E2       GACTTGTTTCCATCATTATAGTCGTAGTATTGATAATTTTGGGAATTTGCGGTTTCTGCA    177
ACUA027427:E2       GGCTCGTGTCCATCATCATCGTGGTAGTACTGATCATACTGGGCGTTTGCGGTTTCTGCA    177
AGAP007942:E3       GGCTCGTGTCTATCATCATCGTCGTAGTACTTATAATTTTGGGCATTTGCGGTTTCTGCA    177
AMEM008390:E3       GGCTCGTGTCTATCATCATCGTCGTAGTACTGATAATTTTGGGCATTTGCGGTTTCTGCA    177
ASTEI07666:E2       GACTCGTGTCCATCATCATCGCCGTAGTACTGATCATTTTGGGCATCTGCGGTTTCTGCA    177
ADIR005979:E3       GACTCGTTTCGATCATCATCGTCGTAGTACTGATAATTCTGGGCATATGCGGTTTCTGCA    177
AFAF002055:E3       GACTAGTTTCGATCATCATCGTCGTAGTACTGATAATTCTGGGCATTTGCGGTTTCTGCA    177
                    * ** ** ** ** ** **     *     * ** **  * **  * ** ** *******

CPIJ004781:E2       TCCGCCGGTGCTTCCGCAAGAGACGCTCCAAAGACGGCAAGAAGGGCATGAAAGGAGTCG    240
AAEL000704:E3       TCCGGCGCTGCTTCCGCAAGAGACGCTCCAAAGATGGCAAGAAGGGCATGAAAGGAGTCG    240
XM_019705510.1      TCCGACGCTGCTTCCGCAAGAGACGCTCCAAAGATGGCAAGAAGGGCATGAAAGGAGTCG    240
AATE010001:E3       TTCGGCGCTGCTTCCGCAAAAGACGATCCAAAGATGGCAAGAAGGGCGCGAAAGGAGTTG    237
ASIC007305:E2       TTCGACGCTGCTTCCGCAAAAGACGATCCAAAGATGGCAAGAAGGGCATGAAAGGAGTTG    237
AEPI006998:E2       TCCGTCGCTGCTTCCGAAAGAGACGATCCAAAGATGGCAAGAAGGGCATGAAAGGAGTTG    237
ACUA027427:E2       TCCGGCGCTGCTTCCGAAAGAGACGGTCCAAAGATGGCAAGAAGGGCATGAAAGGAGTTG    237
AGAP007942:E3       TCCGTCGCTGCTTCCGAAAGAGACGATCCAAAGATGGCAAGAAGGGCATGAAAGGCGTTG    237
AMEM008390:E3       TCCGTCGCTGCTTCCGAAAGAGACGATCCAAAGATGGCAAGAAGGGCATGAAAGGCGTTG    237
ASTEI07666:E2       TCCGGCGCTGCTTCCGAAAGAGACGATCCAAAGATGGCAAGAAGGGCATGAAAGGAGTTG    237
ADIR005979:E3       TTCGGCGCTGCTTCCGAAAGAGACGATCCAAAGATGGCAAGAAGGGCATGAAAGGCGTTG    237
AFAF002055:E3       TTCGGCGCTGCTTCCGAAAGAGACGATCCAAAGATGGCAAGAAGGGCATGAAAGGCGTTG    237
                    * ** ** ******** ** ***** ******** ************  ****** ** *

CPIJ004781:E2       ACCTAAAATCGGTACAATTATTAGGTTCAGCATACAAAGAAAAG       284
AAEL000704:E3       ATCTGAAATCGGTACAATTATTAGGTTCAGCATACAAAGAAAAG       284
XM_019705510.1      ATCTGAAATCGGTACAATTATTAGGTTCAGCATACAAAGAAAAG       284
AATE010001:E3       ATTTGAAATCGGTACAATTATTAGGTTCAGCATACAAAGAAAAG       281
ASIC007305:E2       ATTTGAAATCGGTACAATTATTAGGTTCAGCATACAAAGAAAAG       281
AEPI006998:E2       ATTTGAAATCGGTACAATTATTAGGTTCAGCATACAAAGAAAAG       281
ACUA027427:E2       ATTTGAAATCGGTACAATTATTAGGTTCAGCATACAAAGAAAAG       281
AGAP007942:E3       ATTTGAAATCGGTACAATTATTAGGTTCAGCATACAAAGAAAAG       281
AMEM008390:E3       ATTTGAAATCGGTACAATTATTAGGTTCAGCATACAAAGAAAAG       281
ASTEI07666:E2       ATTTGAAATCGGTACAATTATTAGGTTCAGCATACAAAGAAAAG       281
ADIR005979:E3       ATTTGAAATCGGTACAATTATTAGGTTCAGCATACAAAGAAAAG       281
AFAF002055:E3       ATTTGAAATCGGTACAATTATTAGGTTCAGCATACAAAGAAAAG       281
                    *  * ******************************************
```
